# Preparation and Characterization of Biobased Lignin-Co-Polyester/Amide Thermoplastics

**DOI:** 10.3390/molecules26092437

**Published:** 2021-04-22

**Authors:** Eric L. Young, Armando G. McDonald

**Affiliations:** Department of Forest, Rangeland and Fire Sciences, University of Idaho, Moscow, ID 83844, USA; ericyoung111@gmail.com

**Keywords:** thermoplastics, lignin, lignin- copolyesters/amides, melt-condensation

## Abstract

More than 23 million tonnes of lignin are produced annually in the US from wood pulping and 98% of this lignin is burnt. Therefore, creating products from lignin, such as plastics, offers an approach for obtaining sustainable materials in a circular economy. Lignin-based copolymers were synthesized using a single pot, solvent free, melt condensation reaction. The synthesis occurred in two stages. In the first stage, a biobased prepolymer consisting of butanediol (BD, 0.8–1 molar content) and a diacid (succinic (SA), adipic (AA) and suberic acids (SuA), with varying amounts of diaminobutane (DAB, 0–0.2 molar content) was heated under vacuum and monitored by Fourier transform infra-red (FTIR) spectroscopy and electrospray ionization-mass spectrometry (ESI-MS). In the second stage, prepolymer was mixed with a softwood kraft lignin (0–50 wt.%) and further reacted under vacuum at elevated temperature. Progression of the polymerization reaction was monitored using FTIR spectroscopy. The lignin-copolyester/amide properties were characterized using tensile testing, X-ray diffraction (XRD), dynamic mechanical analysis (DMA), differential scanning calorimetry (DSC) and thermogravimetric analysis (TGA) techniques. Lignin co-polymer tensile (strength 0.1–2.1 MPa and modulus 2 to 338 MPa) properties were found to be influenced by the diacid chain length, lignin, and DAB contents. The lignin-copolymers were shown to be semi-crystalline polymer and have thermoplastic behavior. The SA based copolyesters/amides were relatively stiff and brittle materials while the AA based copolyesters/amides were flexible and the SuA based copolyesters/amides fell in-between. Additionally, > 30 wt.% lignin the lignin- copolyesters/amides did not exhibit melt behavior. Lignin-co-polyester/amides can be generated using green synthesis methods from biobased building blocks. The lignin- copolyesters/amides properties could be tuned based on the lignin content, DAB content and diacid chain length. This approach shows that undervalued lignin can be used in as a macromonomer in producing thermoplastic materials.

## 1. Introduction

Petroleum-based polymers (368 million tonnes/y were produced in 2019) [1] have played a very important role in modern society however, their continued use will consume 25% of current oil production by the end of the century [2]. While the production of many of current polymers requires the incorporation of functional groups (activation) to petroleum-derived precursors, many biomass-derived molecules (e.g., lignin, starch, triglycerides, etc.) already have very reactive functional groups that make them ideal precursors (macro-monomers) for the synthesis of new polymers [3]. The utilization of low or no value lignin streams represents an application of environmentally benign manufacturing that reduces our reliance on fossil fuel, reduces greenhouse gas emissions, and yields a sustainable polymer product. In the USA, more than 23 million tonnes of lignin are produced annually from pulp and paper operations and 98% of this lignin is burnt for energy [4]. To this point, there are two compelling reasons why lignin is not extensively utilized. The first is the present wide availability of relatively low-cost fossil fuel feedstocks. Secondly, the conversion of lignin into a wider range of well-defined products (e.g., plastics) will require the development of new chemical processes that lead to a selective lignin-based products [5].

Lignin is a 3-dimensional biopolymer whose main physiological function is to impart rigidity to the plant cellulosic cell wall for it to stand and to conduct water through the plant vascular system. Its monomeric composition (coniferyl alcohol (guaiacyl unit), sinapyl alcohol (syringyl unit), and *p*-coumaryl alcohol (hydroxyphenol unit)) can vary with plant source (hardwood, softwood, or grass) [6,7]. During wood pulping, lignin is solubilized and undergoes chemical modification such as depolymerization and repolymerization [7,8]. The lignin can be recovered from the pulp liquor (by acid precipitation) to obtain a technical lignin. MeadWestvaco (now WestRock) and West Fraser produce technical lignin from their kraft pulping operations for commercial use. The source of the technical lignin (species and process conditions) will influence its properties, such as glass transition temperature (T_g_), and end uses [9]. 

To utilize technical lignin as thermoplastic materials there have been several approaches namely: (i) blending with other polymers; (ii) modification of lignin to make it thermoplastic or miscible; and (iii) forming lignin-copolymers. Lignin has been successfully blended with a variety of thermoplastics such as polylactic acid (PLA) [10], poly(hydroxybutyrate-co-hydroxyvalerate) (PHBV) [11], polypropylene (PP), poly(ethylene-terephthalate) (PET) [12], and polyethylene oxide (PEO) [13] but acts as a filler. This blending approach has been used to make lignin-based carbon fibers [14]. To produce thermoplastic lignin, the hydroxyl groups on lignin are either esterified [15] or etherified [16]. Esterified (acetates to hexanoates) lignin was also shown to be compatible with PHBV [11]. Esterification is the most feasible approach to modify lignin in terms of the reaction parameters and reactants used [17]. A variety of lignin-co-polymers have been produced such as linear lignin polyester films [18], high-branched lignin-copolyesters with thermo-responsive attributes [9], lignin-copolyurethanes [19] and lignin-coepoxies [20] and this has been extensively reviewed by Glasser et al. [21] and Moreno et al. [22]. There has also been a flurry in research in lignin-grafted-polymers, such as lignin-graft-gum rosin polymers prepared via atom transfer radical polymerization [23]. Chung et al. prepared lignin-graft-PLA by ring opening polymerization catalyzed with triazabicyclodecene [24]. All these copolymer systems have varying degrees of bio-based content. 

To produce fully biobased polyesters and polyamides (nylons), monomeric building blocks (diacids, diols and diamines) need to be bioderived. Organic diacids such as adipic (AA), succinic (SA) and suberic acids (SuA) are now produced via several bio-based routes via metabolic engineering [25]. SA and 1,4-diaminobutane (DAB) can be produced from the fermentation of sugars [26]. 1,4-Butanediol (BD) can be synthesized by direct fermentation of sugars [27]. These building blocks are suitable for making prepolymer polyesters and polyester/amides that can be reacted with lignin (a macromonomer) to form lignin-copolymers. The reactions of AA, SA and SuA with BD will form the following polyesters: poly-butylene-adipate (PBA), polybutylene-succinate (PBS), and polybutylene-suberate (PBSu), respectively. While the reactions of AA, SA and SuA with DAB will form the following polyamides: polytetramethylene-adipamide (PTA), polytetramethylene-succinamide (PTS), and polytetramethylene-suberamide (PTSu), respectively [26].

**Figure 1:**
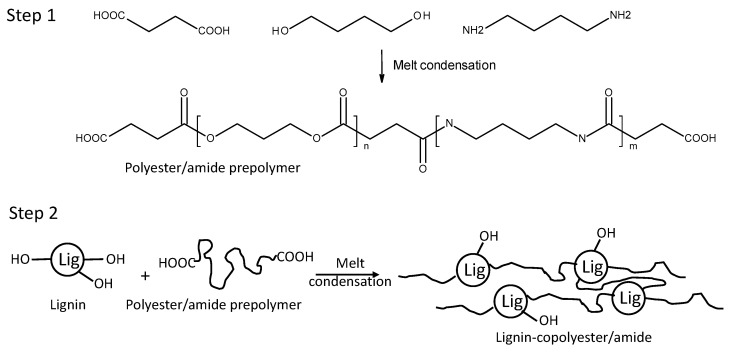
Schematic showing the 2-step process for producing the polybutylene-succinate/succinamide prepolymer and lignin-copolyester/amides (lignin-copolybutylene-succinate/succinamide).

The aim of this study was to produce fully biobased thermoplastic lignin co-polymer using a simple single pot, 2-step process, via a melt condensation reaction (Figure 1). In the first step, a linear low molar mass prepolymer is generated consisting of BD, DAB and one of three diacids (SA, AA, or SuA). Monomer ratios were controlled such that the prepolymer molecules have a higher probability of terminating with carboxylic acid functionality. In the second step, the lignin-copolyester/amides were synthesized by reaction of the lignin hydroxyl groups with the acid groups of the prepolymer. The lignin-copolymers chemical, thermal and mechanical properties were determined to establish their performance as a thermoplastic.

## 2. Results

### 2.1. Prepolymer Preparation

**Figure 2:**
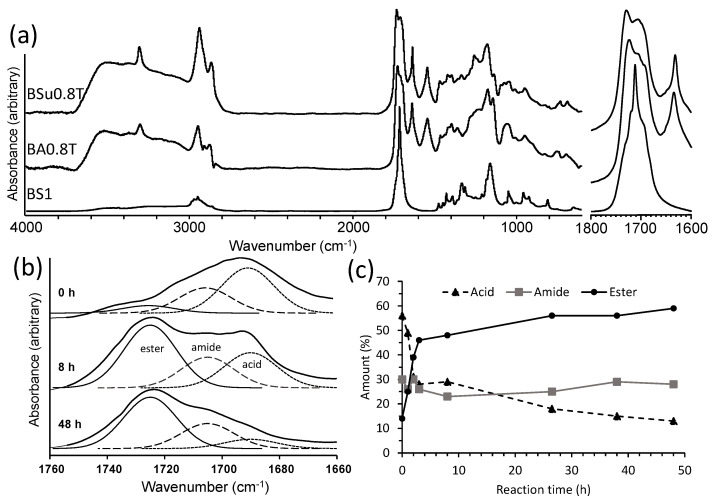
(**a**) FTIR spectra plus expanded carbonyl region of the prepolymers BA0.8T, BS1 and BSu0.8T at 3 h reaction, (**b**) FTIR spectra of the carbonyl region at 0, 8 and 48 h for the prepolymer BA0.8T and (**c**) plot of acid, ester and amide carbonyl stretching bands amount with reaction time for the prepolymer BA0.8T.

A range of prepolymers were prepared using a various diacids (AA, SA, SuA), BD and DAB at differing proportions, via a melt condensation reaction (Table 1, Figure 1). Nomenclature for the specific prepolymer formulations are given in Table 1. To eliminate the use of solvents, following a “green chemistry” approach, BD was used as a reactive solvent. Fourier transform infra-red (FTIR) spectroscopy was used to monitor (0–48 h) the formation of ester and amide functional groups in the prepolymer during the reaction. FTIR spectra for prepolymers BA0.8T, BS1, and BSu0.8T after 3 h reaction are shown in Figure 2a. For the polyesters two bands were observed, while the polyester-amide three separate bands were observed at 1690, 1705 and 1725 cm^−1^ for the carboxylic acid, amide and ester carbonyl stretch, respectively [5,28]. The bands were fitted to determine acid, amide and ester contents (Figure 2b). For BA0.8T prepolymer, a plot showing ester and amide linkage formation with reaction time is shown in Figure 2c. This clearly showed the progression of ester and amide bond formation during the pre-polymerization reaction. The amide band remained relatively constant throughout the reaction suggesting that DAB reacted quickly [5,28]. As reaction time progressed the prepolymer changed from a liquid to a solid (>4 h), therefore the prepolymer reaction time of 3 h was chosen for copolymerization. 

**Figure 3:**
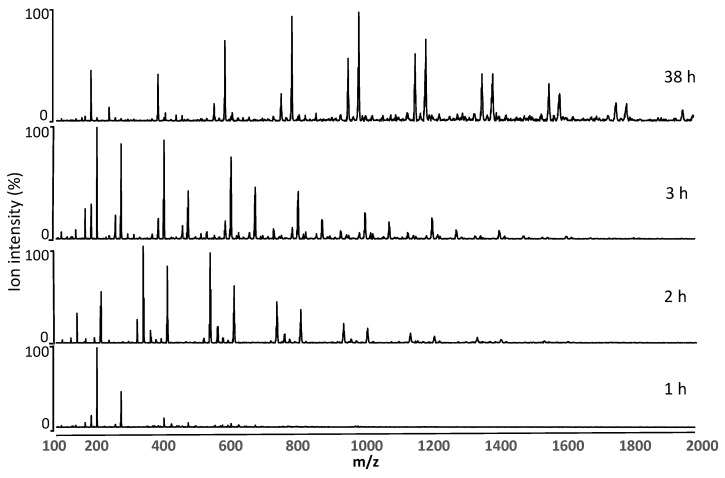
Positive ion ESI-MS of prepolymer BA0.8T at 1, 2, 3 and 38 h of polymerization.

Electrospray ionization mass spectrometry (ESI-MS) was used to monitor oligomer progression during the prepolymerization reaction (Figure 3) and determine the M_n_ and M_w_ of the various prepolymer formulations at 3 h and 48 h of reaction (Table 2) [28]. The SA based prepolymers at 48 h were insoluble in CHCl_3_, CH_3_OH and tertahydrofuran (THF) and could not be analyzed by ESI-MS. The mass spectra of the prepolymer BA0.8T from 1 to 38 h of polymerization are shown in Figure 3. The most common ions detected were [M + H]^+^, [M + Na]^+^, and [M − OH]^+^ adducts. Nearly all the peaks were able to be identified through this type of analysis. For the BA0.8T prepolymer, the [M −OH]^+^ ions at m/z 199, 269, 399, 600, 728 and 799 were assigned to AA + DAB, AA + 2BD, 2AA + DAB + 1BD, 3AA + DAB + 2BD, 4AA + DAB + 2BD, and 2AA + 2DAB + 2BD oligomers, respectively. A series of [M + Na]^+^ ions at m/z 619, 968, and 1168 were assigned to 3AA + 3BD, 5AA + DAB + 3BD and 6AA + DAB + 4BD oligomers, respectively. This provides further evidence for the progression of the polymerization reaction with time. 

The melting temperature (T_m_) of the prepolymers after 48 h reaction were determined by differential scanning calorimetry (DSC) and dynamic mechanical analysis (DMA) using parallel plate compression (Table 3). The T_m_ values for the polyester prepolymers was about 44 °C for the BA and BSu based prepolymers and 100 °C for the BS prepolymer. The T_m_ values determined by DMA were comparable to those determined by DSC. The addition of DAB to the BS and BA based prepolymer slightly increased its T_m_, while a greater increase (20 °C) was observed for the BSu prepolymers. 

### 2.2. Lignin-Co-Polyester-Amide Preparation

**Figure 4:**
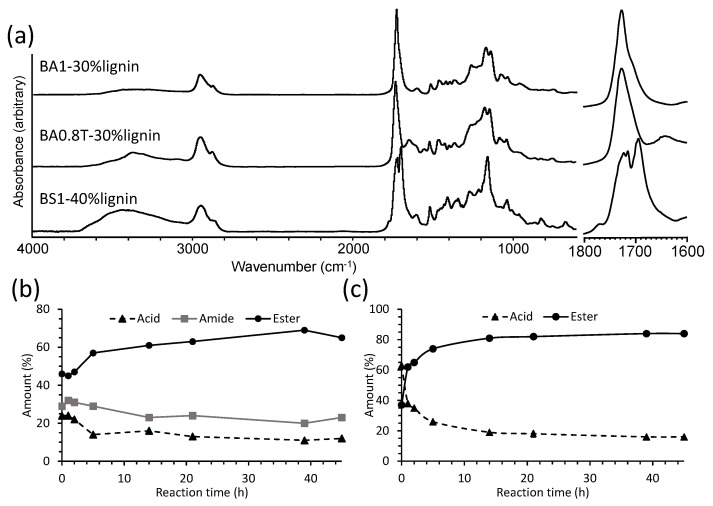
(**a**) FTIR spectra plus expanded carbonyl region of BA0.8T-30%lignin, BA1-30%lignin and BS1-40%lignin copolymers after reaction for 45 h and plots of acid, ester and amide carbonyl content determined by FTIR spectroscopy with reaction time for the lignin-copolymer (**b**) BA0.8T-30%lignin and (**c**) BA1-30%lignin.

The prepolymer reaction time of 3 h was selected for lignin copolymerization because it was still in the liquid state. The lignin-copolymers were synthesized by first dispersing the lignin in the liquid prepolymer and subsequently reacted by melt condensation in a one-pot process for 45 h (Figure 1). The influence of prepolymer composition (diacid type, DAB content) and 0–50% lignin content on lignin-copolymer properties (Table 1) were examined. The lignin-copolymers were prepared into sheets ranging from flexible to brittle materials and had a dark brown appearance. A photograph of several lignin-copolyester/amide specimens is shown in Figure S1. The lignin-copolymer reaction was monitored by FTIR spectroscopy (Figure 4a). For the polyester/amide based copolymers, amide I and II bands were observed at 1650 cm^−1^ and 1546 cm^−1^, respectively [28]. For all lignin-copolymers, the band at 1150–1170 cm^−1^ was assigned to C-O stretching in esters [28]. The C=O carbonyl stretching band for acids, esters, and amides was seen at 1680–1780 cm^−1^ [28]. The broad band at 3000–3600 cm^−1^ was assigned to O-H and N-H stretching. C-H vibration bands associated with asymmetric and symmetric stretching for CH*_2_* stretching were observed at 2950 and 2880 cm^−1^, respectively [11]. Two distinctive lignin aromatic skeletal vibrations at 1515 and 1600 cm^−1^ were clearly observed [9]. Peak fitting of the carbonyl band was used to determine the proportions of ester, acid and amide contents [5,28,29]. Figure 4b and c shows the plot of acid, ester and amide contents with reaction time for the lignin-copolymers BA0.8T-30%lig and BA1-30%lig, respectively. The ester content increased to 70% (at 40 h) while the amide was about 20% in the BA0.8T-30%lig copolymer. For the lignin-copolyester (BA1-30%lig) the ester content reached 84% at 45 h. The other lignin-copolymer formulations showed the same behavior during the polymerization reaction (data not shown). 

### 2.3. Thermal Analysis of Lignin-Co-Polyester-Amides

**Figure 5:**
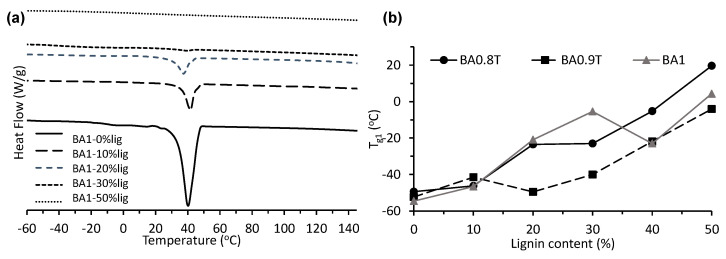
(**a**) DSC thermograms (1st heating cycle) of BA1-0-50%lignin copolymers (endotherm down) and (**b**) plot of glass transition temperature (T_g1_) versus lignin content for BA1, BA0.8T, BA0.9T lignin-copolymer.

The thermal properties (T_g_ and T_m_) of the various lignin-copolymer formulations were determined by DSC (Figure 5a, Figures S2–S10, and Table 4). Figure 5a shows DSC thermograms of BA1-0–50%lignin copolymers on the first heating cycle. The first T_g_ (T_g1_), weak signal, was assigned to the amorphous polyester/amide regions [18,26] of the lignin-copolymer which ranged between −54 to 20 °C for the BA-lignin copolymers, between −29 to 23 °C for the BS-lignin copolymers, and between −46 to 8 °C for the BSu-lignin copolymers. Figure 5b shows a plot of T_g1_ versus lignin content for AA-BD lignin copolymers. A second T_g_ (T_g2_) was observed in most of the lignin-copolyester/amide samples between 116 and 126 °C (Table 4). Polymer samples containing 0% lignin did not exhibit this T_g2_ transition. This transition was close to that of softwood kraft lignin T_g_ at 143 °C [9]. An endothermic T_m_ peak was observed for the lignin copolymers up to a lignin content of 30% which supports that these were semi-crystalline materials (Table 4). The presence of a cold crystallization exothermic peak (−20 to 90 °C) was also observed in many of the thermograms for the BA, BS and BSu based lignin-copolymers (≤20% lignin) which also supports the semi-crystalline nature of these lignin-copolymers (see Figures S2–S10).

**Figure 6:**
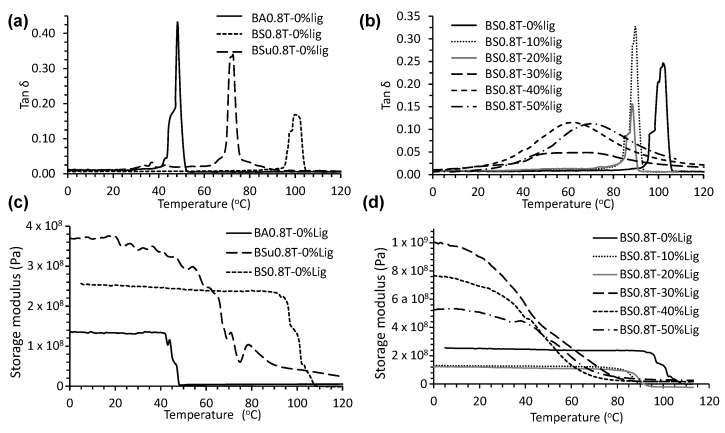
DMA tan δ thermograms of (**a**) polymers BA0.8T, BS0.8T and BSu0.8T, (**b**) BS0.8T-0−50%lignin copolymers, and storage modulus thermograms of (**c**) polymers BA0.8T, BS0.8T and BSu0.8T, and (**d**) BS0.8T-0−50%lignin copolymers.

The thermal properties (such as T_m_) of the various lignin-copolymer formulations were also determined by DMA, using the tan δ signal [30] (Figure 6a,b and Table 5). The T_m_’s ranged from 38 to 103 °C depending on lignin-copolyester/amide composition. Figure 6 shows DMA thermograms of polymers BA0.8T, BS0.8T and BSu0.8T and BS0.8-0−50%lignin copolymers. The T_m_’s determined by DMA were comparable to those determined by DSC. The storage moduli (E’) at 15 °C for all the lignin-copolymers were also determined by DMA (Figure 6c,d, Table 5). The storage moduli ranged between 88 and 3390 MPa.

**Figure 7:**
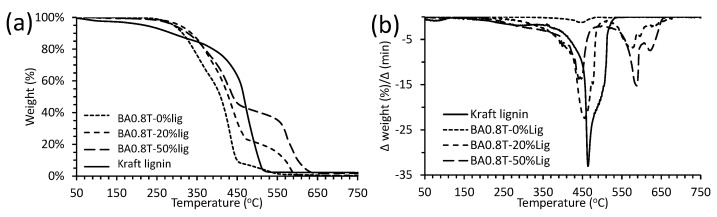
(**a**) TGA and (**b**) DTG thermograms of kraft lignin and BA0.8T-0, 20, 50%lignin copolymers.

The thermal stability of the lignin-copolyester/amides were determined by thermogravimetric analysis (TGA) and differential thermal gravimetry (DTG). Table 6 summarizes the 1st major degradation T_onset_ and the T_d5_ values of the various lignin-copolyester/amides. The T_d5_ values were generally lower than the major T_onset_ values. The TGA and DTG thermograms for BA0.8T-lignin copolymer series and kraft lignin are shown in Figure 7. Lignin degradation was shown to occur in 3 stages. The first temperature onset (T_onset_, ~100 °C) is due to water loss [31]. The second T_onset_ (~210 °C) is attributed to degradation of residual carbohydrate and the final T_onset_ near 420 °C with most of the weight loss is attributed to lignin degradation [9]. 

### 2.4. Crystallinity of Lignin Copolymers by XRD

**Figure 8:**
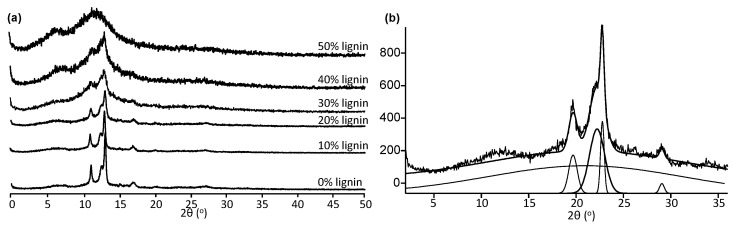
X-ray diffractograms of (**a**) BS0.8T-0−50%lignin copolymers and (**b**) BA1-20%lignin copolymer showing peak fitting.

The crystalline content of the various lignin-copolymer formulations was determined by X-ray diffraction (XRD) after peak fitting [32] (Figure 8 and Table 7). Figure 8a shows X-ray diffractograms of the BS0.8T-0–50%lignin copolymers and Figure 8b show a peak fitted diffractogram of BS1-20%lignin copolymer. It can be seen in the diffractograms of the lignin-copolymers that the crystallinity decreased (with a concomitant increase in the amorphous content) from 48% to 5% with lignin content. 

### 2.5. Tensile Properties of Lignin Copolymers

**Figure 9:**
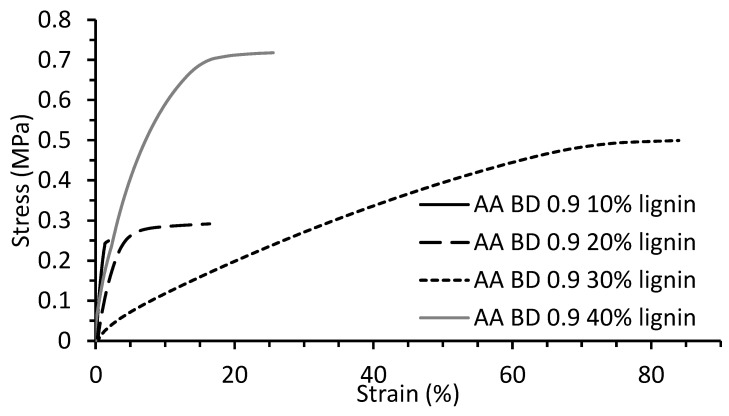
Tensile stress-strain curves of BA0.9T-10–40%lignin copolymers.

The tensile properties of the various lignin-co-polyester/amides sheet samples were determined. Figure 9 shows the stress strain curves of BA0.9T-10−40% lignin copolymers. The tensile strength, Young’s modulus, and strain at break and associated standard deviations for the various lignin-copolyester/amides are given in Table 8. For the various lignin-copolyester/amide series the average tensile modulus spanned from 2 to 338 MPa, the average tensile strength ranged between 0.09 to 2.11 MPa, and the average strain at break spanned between 0.7 and 73%. 

## 3. Discussion

### 3.1. Prepolymer Preparation

The various linear prepolymers (BAT, BST, and BSuT) formulations were prepared using either AA, SA or SuA diacids together with BD and DAB by melt condensation. A “green chemistry” approach was used (solvent free) by using BD as a reactive solvent [31]. This approach has been used before in making highly branched prepolymers in which glycerol was used as the reactive solvent [28]. At 4 h reaction time, the prepolymer became a solid and was not suitable for subsequent copolymerization. Therefore, a prepolymer reaction time of 3 h was selected since it was in a liquid state and could disperse lignin. FTIR spectroscopy was used to examine the chemical structural feature of the polyester/amide prepolymers. Spectra for BA0.8T, BS1, and BSu0.8T after 3 h reaction are shown in Figure 2a. Two bands at 1650 cm^−1^ and 1555 cm^−1^ in the DAB containing prepolymers were assigned as amide I and amide II bands, respectively [28]. For all prepolymers, a band at 1150–1170 cm^−1^ was assigned to C-O stretching in esters [28]. Broad bands with high intensity from 3500 to 3000 cm^−1^ were due to high amounts of OH and COOH groups present. C-H vibration bands associated with asymmetric and symmetric stretching for CH*_2_* stretching were respectively observed at 2950–2900 and 2850–2880 cm^−1^ [11]. FTIR spectroscopy of the prepolymer reaction clearly showed the progression of ester and amide bond formation and DAB reacted quickly (Figure 2) [5,28,29]. ESI-MS was employed to monitor the prepolymerization reaction and determine its molar mass (Table 2). At 3 h reaction time, the M_n_ for the various prepolymer formulations ranged between 406 and 927 g/mol and deemed a useful size for subsequent co-polymerization with lignin [29]. 

The 48 h-reacted solid PBS had a higher T_m_ ~100 °C than PBA and PBSu (~44 °C) and this is likely due to its short chain length (C_4_ vs. C_6_ and C_8_) contributing as a hard segment [18]. Incorporating DAB into the prepolymer also increased its T_m_. Work by Wang et al., on poly(ester-amines) showed that T_g_ values decreased as DAB content was increased [33]. 

### 3.2. Lignin-Co-Polyester/Amide Preparation

Softwood kraft lignin has suitable attributes (molar mass (93,700 g/mol) [11], hydroxyl content (1.08 OH/C_9_OCH_3_ units) [16], and T_g_ (143 °C) [9]) as a hard segment in copolymers. Lignin was readily dispersed in the 3 h reacted prepolymer formulations and subjected to a “green chemistry-solvent free” melt condensation reaction to form copolymer sheets [34]. FTIR spectroscopy (Figure 4) clearly showed both ester and amide bands being formed in the lignin-copolyester/amides and generally their content increased over the 45 h reaction period, respectively, at 70% and 20%. For the lignin-copolyesters an ester content of 85% was generally achieved. These results indicates that the lignin is reacting with the excess acid groups in the prepolymers to form the lignin-copolymers. This has been observed in highly branched lignin-copolymers [5,28,29].

### 3.3. Thermal and XRD Analyses of Lignin-Co-Polyester/Amides

The various lignin-copolyester/amides were shown to be semicrystalline thermoplastics with having two T_g_’s and a T_m_ as determined by DSC (Figure 5 and Figures S2 to S10 and Table 4). The amorphous polyester/amide regions of the lignin-copolymer were assigned to the weak T_g1_ by DSC (−54 to 20 °C for BA-copolymers; −29 to 23 °C for BS-copolymers; −46 to 8 °C for BSu-copolymers) [18,26]. Literature reported T_g_’s for PBS and PBSu were -34 °C [35] and −60 °C [36], respectively. Generally, the T_g1_ increased and broadened with lignin content suggesting that lignin reinforced the copolyester/amide by linking and/or associations between the two. The DSC determined T_g2_ (between 116 and 126 °C) was close to lignin T_g_ at 143 °C (Table 4). It is therefore speculated that this transition is attributed to lignin [9]. The presence of this transition also suggests incomplete polymerization, without the use of catalyst, and the presence of a heterogeneous material [37]. The lignin-copolymers (up to 30% lignin content) showed a T_m_ which supports the XRD data of a semi-crystalline material (Table 4 and Table 7). An increase in lignin content was shown to reduce the crystallinity of the lignin-copolymers. DSC analysis of the lignin-copolymers showed a similar melt behavior on the second heating cycle confirming melt behavior, notably at 10 and 20% lignin contents but in some cases at 30% lignin (Figures S2–S10). The BS0.8T, BS0.9T and BS1 lignin copolymers had T_m_ ranging from 78 to 105 °C, the 48 h cured prepolymers were 103–106 °C and these were lower than reported for PBS at 114 °C [38]. The BA0.8T, BA0.9T and BA1 48 h cured prepolymers and lignin copolymers had T_m_’s ranging from 27 to 40 °C and were lower than for PBA (~50 °C) [39]. The T_m_ for BSu0.8T, BSu0.9T and BSu1 48 h cured prepolymers and lignin copolymers were between 32 to 45 °C and slightly lower than reported for PBSu (~54 °C) [36]. The DAB content did affect the T_m_ such that the BA0.8T-lignin copolymers gave the lowest T_m_ of 27 °C and the BA1-lignin copolymers gave the highest T_m_ at 40 °C, by DSC (Table 4). In other words, the presence of DAB in the copolymer reduced the T_m_. The BST lignin copolymer series showed a T_m_ decrease with lignin content [40]. In addition, the T_m_ peak decreased in size with lignin content. For the lignin-copolyester/amides at 40 and 50% lignin content the absence of a melt transition for the polyester/amide component suggests that lignin inhibits crystallization. 

DMA was also used to determine the T_m_ of the various lignin-copolymers (Figure 6 and Table 5) and the T_m_ values corresponds with a melt observed by DSC. T_m_ values, determined by DMA, for the BAT-lignin copolymers were shown to span from 38 to 82 °C. Most of the increase in T_m_ occurs at lignin contents > 30 wt.%. At lignin contents < 30 wt.% the values for T_m_ were around 45 °C. The addition of DAB to the BA-lignin copolymers generally increased its T_m_ and at 0.2 mole ratio DAB gave the best performing lignin-copolymers. Furthermore, the lignin-copolymer’s T_m_ could be tuned from 38 to 82 °C by varying the amount of lignin (10 to 50%). This has been observed in other lignin-copolymers [41]. The T_m_ values for the BSuT-lignin copolymers follow a similar trend to the BAT-lignin copolymers and range from 40 to 86 °C. The increase in T_m_ occurs at lignin contents > 30 wt.%. In the case of the BSU0.8T-lignin copolymers the T_m_ decreased from 68 to 40 °C upon increasing the lignin from 0 to 10 wt.%. At 50 wt.% lignin for the lignin-copolymers a T_m_ value of 86 °C was achieved. The BST-lignin copolymers had a T_m_ between 55 and 103 °C with the highest T_m_ value a from the BS0.9T-0%lignin copolymer. The BST-lignin copolymers showed a decrease in T_m_ with increasing lignin content, in contrast to the BAT and BSuT copolymers. In contrast to the BAT and BSuT copolymers, was the observation that the BSu0.8T-lignin copolymers gave the lowest T_m_ at 10 to 50 wt.% lignin. The BST-0%lignin copolymer had a T_m_ of 102 °C which was considerably greater than T_m_’s for the BSuT (55 °C) and BAT (45 °C) based polymers. This demonstrates that the BST prepolymer molecules experience greater intermolecular attractive forces than BSuT and BAT based polymers [12,13]. The reduced molecular mobility also reduces the dampening effect which can be seen by comparing the height of the tan δ signals for the BA0.8T-0%lig and BSu0.8T-0%lig based polymers (Figure 6a). The tan δ signal for the BS0.8T-0%lig sample was less intense than what is observed for the BA0.8T-0%lig and BSu0.8T-0%lig polymers which supports the concept of reduced molecular mobility in SA based polymer [41]. The reduced mobility may be due to the lignin-copolymer being slightly branched due to the numerous hydroxyl groups in lignin limiting its mobility. This effect also has implications for the mechanical properties.

The BAT and BSuT lignin copolymer series generally showed an increase in T_m_ by DMA with lignin content (Table 5) and this has been reported in the literature [40,41]. This shows that lignin helped reinforce (hard segment) the copolymer network (soft segment). However, the BST lignin copolymer series showed a decrease in T_m_ with lignin content. As lignin content increases the amorphous nature of the material also increases as shown by XRD (Figure 8). This behavior is partly attributed to the amorphous nature of lignin and disruption of crystal formation within the lignin-copolymer structure [32]. However, this alone does not explain the very low levels of crystallinity observed at the 40 and 50 wt.% lignin levels. It is speculated that the lignin structure also reduces crystallinity by interrupting the crystalline nature of the polyester/amine zones within the copolymer. Similar findings have been observed in other copolymer systems [24]. 

The E’ was measured for all the lignin-copolymers at 15 °C for comparative purposes by DMA (Table 5). The lowest E’ was for BA1-50%Lig at 88 MPa and the highest E’ was for BSu0.9T-0%Lig at 3.4 GPa. For comparison of the polymers without lignin at 15 °C, BSu0.8T had the highest E’ at 371 MPa as compared to BS0.8T at 253 MPa and BA0.8T at 195 MPa. Figure 6d shows the thermograms of BS0.8T with lignin contents varying from 0 to 50% lignin and clearly show a drop in E’ signal as 10 and 20% lignin is incorporated into the copolymer, then increases with incorporation of further lignin > 30%. This shows that lignin was reinforcing the material. No obvious trends between composition and E’ were lignin-copolymers were observed.

TGA was used to determine the degradation behavior of the lignin-copolymers and occurs in three stages (Figure 7). The first two stages are associated with the polyester/amide section, while the 3^rd^ stage is attributed to lignin between 480 °C and 640 °C. The weight difference between the last stage correlates well with the lignin content [5]. For example, the BA0.8T-0%lig polymer exhibits two degradation stages: the first and major T_onset_ occurs at 360 °C and is attributed to the ester linkages [5] and the second T_onset_ occurred at 420 °C and attributable to amide linkages. Lignin was shown to improve the thermal stability of the lignin-copolymers and this has been observed in lignin-copolyurethanes [42]. In all cases the main T_onset_ increases from the neat prepolymer to the 10 wt.% lignin level (Table 6). This contrasts with lignin-copolymers in a previous study which exhibited diminished onset values upon the addition of lignin to the prepolymer [5]. It was observed for the 50% lignin content lignin-copolymers that the T_onset_ decreased, and this behavior is consistent with cited research [5]. An interesting observation was the lowest T_onset_ values, in many cases, was attributed to the highest level of DAB. To illustrate this, the BST-30%lig copolymers had a decrease in onset temperatures from 421 °C to 346 °C and finally to 327 °C in order of increasing DAB content from 0 to 0.1 to 0.2 mole ratio, respectively. The T_d5_ values were also determined and were lower in value than the major T_onset_ values for each of the lignin-copolymers. It was originally hypothesized that the thermal stability of the lignin-copolymers would be enhanced upon addition of amide hydrogen bond donating groups, but this does not appear to be the case. Depending on the copolymer formulation with respect to lignin and DAB content the thermal degradation T_onset_ can be adjusted through a range from 292 °C to 431 °C.

### 3.4. Tensile Properties of Lignin Copolymers

**Figure 10:**
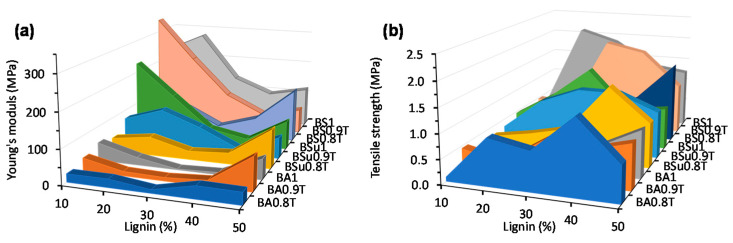
Plot of (**a**) Young’s modulus versus lignin content and (**b**) tensile strength versus lignin content for the various lignin-copolyester/amide formulations.

The tensile properties (strength, Young’s modulus, and strain at break) of the lignin-co-polyester/amides were determined and given in Table 8. The modulus for the BAT-lignin copolymer series spans from 2 to 97 MPa, the BST-lignin copolymer series ranged between 23 and 338 MPa, and the BSuT-lignin copolymer series ranged between 16 and 133 MPa (Figure 10a). The observed Young’s modulus was in the range of LDPE at 100–200 MPa [43]. The shorter chain length of SA versus AA makes for a harder polymer. While the longer chain length of SuA versus SA makes it more flexible. Generally, the modulus of the BAT-lignin and BST-lignin copolymers decreased to a minimum with an increase in lignin content at 30% lignin. At 40 and 50% lignin the modulus increased and then increased. One possible explanation for this behavior arises from the morphological characteristics of the copolymer. At low lignin loading (0–20%) the crystalline nature of the prepolymer is the dominant contributor to the morphology which endows a high modulus and a low capacity for strain. At 30 wt.% lignin the lignin-copolymer is more ductile thus increasing free volume in the matrix and allowing for more deformation with less stress than at lower and higher lignin loadings. This phenomenon has been observed in other lignin-polymers [13]. At 40–50 wt.% lignin content the brittle and stiff nature of the lignin (highly packed) begins to dominate the copolymer characteristics. The exception is the case of the BA0.8T-lignin copolymers at 40 and 50 wt.% lignin contents had comparable moduli. Similar results have been observed in which incorporation of a chain-extended hydroxypropyl lignin into a polymethyl methacrylate prepolymer matrix at increasing lignin contents led to a lower modulus while incorporation of a crosslinked lignin derivative raised the modulus [13].

Originally it was hypothesized that by increasing the prepolymer DAB content would result in a higher modulus polymer due to an increase in hydrogen bonding. This behavior is observed for the 20, 30 and 40 lignin wt.% lignin-copolyester/amide. However, at the 10 and 50 wt.% lignin levels the opposite effect is true. That is the stiffest material is the copolymer containing no DAB. This behavior is counter-intuitive, and an explanation is not immediately apparent. 

The tensile strengths (Table 8, Figure 10b) for the BAT series of lignin-copolymers were shown to span a range from 0.09 MPa for BA0.8T-10%lig to 1.4 MPa BA0.8T-40%lig. The effects of amine content on Young’s modulus were generally mirrored in the tensile strength data. At both the 10 and 50 wt.% lignin levels the 0.8 mole ratio BD lignin-copolyester/amides showed a diminished tensile strength as compared to their counterparts having lower amine contents. At the 20, 30 and 40 wt.% lignin levels, however, the tensile strength values were shown to reverse this trend with the highest tensile strength value belonging to the lignin-copolymers with 0.2 mole DAB and the lowest tensile strength value attributed to the lignin-polyester copolymers (0 mole ratio DAB). The observed tensile strength values were lower than for LDPE at 4-10 MPa [43]. Furthermore, the strain at break values (ranged from < 1 to 70%) were also considerably less than LDPE (90–800%).

Qualitative melt testing on the lignin-copolymers with lignin content ≥30% were shown not to exhibit melt behavior. This observation supports the DSC results in which no melting endotherms were observed for copolymers at 40% and 50% lignin content. In addition, melting behavior was mainly observed for lignin-copolymers with 10% and 20% lignin, except for BA1-20%lignin. These results suggest that at high lignin content has an effect of reducing the mobility of the copolymer molecules and ultimately leads to a material that is incapable of melting. In addition, the lignin-copolymer may be slightly branched due to the numerous hydroxyl groups in lignin limiting its mobility. 

### 3.5. Structure of Lignin-Copolymers

**Figure 11:**
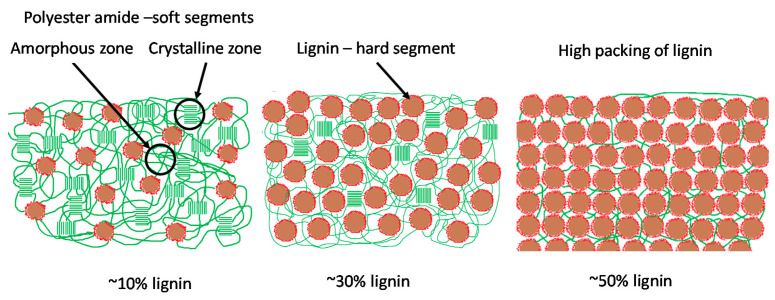
Schematic representation of the structure and packing of the lignin-copolyester/amides at 10, 30 and 50% lignin content.

At low lignin contents (0–20 wt.%) the DSC and DMA response are attributed to the melting of the crystalline regions of the prepolymer. However, at the 30 wt.% lignin level the melting behavior ceases in the DSC analysis (Figure 5 and Figures S2–S10) but strongly observed in DMA (Figure 6). The amorphous regions of the copolymer within the lignin-copolymer results in the T_g1_ transition. Therefore, T_g1_ by DSC is observed to increase with lignin content. The system behaves as a blend. It is postulated that the DMA response beginning at 30 wt.% lignin is the result of the T_11_ transition [44]. This T_11_ transition is observed in amorphous materials and can be attributed to the breakdown of segment-segment associations which increases segment mobility [44]. These segments are the lignin aggregates (hard segment) with the association regions being represented by random polymer chains (soft segment) surrounding the lignin aggregates. For the 10 wt.% lignin case the random polymer zone associated with the amorphous T_11_ is not in close enough proximity to another lignin aggregates to develop interactions (Figure 11). However, as lignin content is increased these interactions become the dominant attractive force as shown in the representation of the 50 wt.% lignin (Figure 11).

## 4. Materials and Methods

Softwood kraft lignin (Indulin AT) was obtained from Mead Westvaco (Charleston, SC, USA) Succinic acid (SA) was obtained from Mallinckrodt Chemical Works (Bedminster, NJ, USA). Adipic acid (AA) and 1,4 diaminobutane (DAB) were obtained from Acros Organics (Morris Plains, NJ, USA) and suberic acid (SuA) and 1,4 butanediol (BD) were all obtained from Alfa Aesar (Ward Hill, MA, USA). 

The prepolymer was prepared by mixing a 1.1 molar ratio of diacid together with the desired molar ratio of BD and DAB such that the sum of the molar ratios of BD + DAB was 1 relative to the diacid [5]. The levels of DAB tested were 0, 0.1 and 0.2 molar content. For the 0 wt.% lignin samples diacid was present at a molar ratio of 1. The prepolymer mixture was stirred thoroughly and placed in a vacuum oven at −50 KPa and 60 °C for 30 min. The mixture was then stirred again and placed in the vacuum oven at −50 KPa and 140 °C for a total of 3 h. Examples of sample names with their corresponding chemical makeup are provided in Table 1. 

For preparing the lignin-copolymers, lignin was added to the prepolymer at mass ratios of 0, 10, 20, 30, 40 and 50 wt.% and the mixtures were stirred thoroughly and placed in Teflon molds. The molds containing the lignin-copolymer mixtures were then placed in a vacuum oven at 150°C and −50 KPa for 45 h. 

FTIR spectra of the prepolymers and copolymers were obtained using an iS5 spectrometer (Thermo-Nicolet, Madison, WI, USA) equipped with a ZnSe attenuated total reflection (iD5 ATR) accessory. Peak fitting analysis was performed using Igor Pro version 6.03. Molar masses of the prepolymer samples (1 mg/mL in 1:1 CHCl_3_/CH_3_OH containing 1% CH_3_COOH) were determined by positive ion ESI-MS using a Finnigan LCQ-Deca (ThermoQuest, San Jose, CA, USA) instrument. Spectra were from mass to charge (m/z) ratio range of 80–2000. The number and weight average molar mass were calculated respectively as M_n_ = ∑N_i_M_i_/∑N_i_ and M_w_ = ∑N_i_M_i_^2^/∑N_i_M_i_, where N_i_ is the intensity and M_i_ is the mass after accounting for the charge. The polydispersity index (PDI) was calculated as M_w_/M_n_ [28]. X-Ray diffraction (XRD) was performed on copolymer samples using a D5000 diffractometer (Siemens, Carteret, NJ, USA) using a Cu Kα source (1.542 Å) running at 40 kV from the 2θ range of 2° to 50° at 0.5° increments. The crystallinity index was determined using a diffraction deconvolution method with the peak positions and width held constant using IgorPro v6.03 software [45]. The ratio of crystalline peaks to total peak area including amorphous peaks was used to calculate percent crystallinity. 

DSC was performed on a Q200 DSC (TA Instruments, New Castle, DE, USA) equipped with a refrigeration unit. To determine the polymers T_g_’s, the samples were first annealed at 135 °C for 1 min to erase any thermal memory [9]. Samples were then analyzed from −70 to 150 °C at a heating rate of 5 °C/min with a temperature modulated (TMDSC) profile overlaid of + 0.5 °C every 20 s. The T_g_s were recorded as the temperature associated with the minimum value of the first derivative of the heat flow signal at the steepest point in the inflection found in the thermogram showing heat flow versus temperature. TGA was performed on a TGA-7 instrument (Perkin Elmer, Shelton, CT, USA) from 30 to 900 °C at a heating rate of 20 °C/min. Thermal stability was determined at the onset (T_onset_) of thermal degradation from two tangent lines as well as the temperature at 5% weight loss (T_d5_). DMA was performed on a Perkin Elmer DMA 7 instrument in compression mode from −20 °C to 140 °C at a heating rate of 5 °C/min using a static force of 20 mN and a dynamic force of 16 mN at 1 Hz. The temperature associated with the peak maximum in the tan δ signal is reported as the T_g_ [30]. 

Tensile testing was performed on a TA Q800 DMA instrument at 30 °C on rectangular (7 mm × 27 mm) specimens using a force ramp of 1 N/min to 18 N [41].

Qualitative melt testing was performed on lignin-copolymer samples (2 g) between 2 pieces of parchment paper and heated to 115 °C for 10–15 min in a laboratory hot press (300 mm × 300 mm, PHI, South El Monte, CA, USA). Melting behavior was assessed whether the sample exhibited any flow behavior. 

## 5. Conclusions

Lignin-copolyester/amides were generated through a green synthesis method that avoided the use of solvent. The melt condensation synthesis route was observed to be relatively simple. Additionally, the resulting copolymer utilized up to 50% kraft lignin, an abundantly available, renewable polymer as well as other monomers with potential renewable sourcing. The lignin-copolymers were shown to be semicrystalline thermoplastics and the crystallinity decreased with lignin content. The lignin-copolymer showed thermoplastic melt behavior with lignin content only up to 20%. The copolymers exhibited a range of mechanical and thermal properties that demonstrated the ability to be tuned based on the lignin content, DAB content and type of diacid used. DMA revealed the presence of T_11_ thermal transitions at > 30 wt.% lignin and are likened to a quasi-melt transition occurring within the amorphous regions where the association of lignin aggregates broke down and allowed for greater mobility. The BST-lignin copolymers were observed to be relatively stiff, brittle materials while the BAT-lignin copolymers were more flexible (elastomers). The observed Young’s modulus values ranged from 5 MPa to 340 MPa and were comparable to natural rubber and other elastomers at 100 MPa. However, the tensile strengths were lower than that of natural rubber. Additional work is required to improve the properties of these lignin-copolymers. This could be achieved by use of an acid catalyst (*p*-toluene sulphonic acid) to speed up (from 45 h to 3 h) and encourage the polymerization reaction to form the copolymer linkages and increase molar mass. 

## Figures and Tables

**Table 1:** Prepolymer and lignin-copolymer formulations with monomer composition and lignin content.

Sample Name	Acid	BD (Mole Ratio)	DAB (tetramethylene (T)) (Mole Ratio)	Acid (Mole Ratio)	Lignin Content (%)
BS0.8T-0%lig	SA	0.8	0.2	1	0
BS0.8T-10–50%lig	SA	0.8	0.2	1.1	10–50
BS0.9T-0%lig	SA	0.9	0.1	1	0
BS0.9T-10–50%lig	SA	0.9	0.1	1.1	10–50
BS1-0%lig	SA	1.0	0	1	0
BS1-10–50%lig	SA	1.0	0	1.1	10–50
BA0.8–0%lig	AA	0.8	0.2	1	0
BA0.8-10–50%lig	AA	0.8	0.2	1.1	10–50
BA0.9–0%lig	AA	0.9	0.1	1	0
BA0.9-10–50%lig	AA	0.9	0.1	1.1	10–50
BA1-0%lig	AA	1.0	0	1	0
BA1-10–50%lig	AA	1.0	0	1.1	10–50
BSu0.8–0%lig	SuA	0.8	0.2	1	0
BSu0.8-10–50%lig	SuA	0.8	0.2	1.1	10–50
BSu0.9–0%lig	SuA	0.9	0.1	1	10–50
BSu0.9-10–50%lig	SuA	0.9	0.1	1.1	10–50
BSu1-0%lig	SuA	1.0	0	1	0
BSu1-10–50%lig	SuA	1.0	0	1.1	10–50

**Table 2:** Prepolymer M_n_, M_w_ and PDI determined by ESI-MS at 3 h and 48h.

	M_n_ (g/mol)			M_w_ (g/mol)			PDI		
	SA	SuA	AA	SA	SuA	AA	SA	SuA	AA
**3 h**									
BD0.8	406	552	927	715	763	1140	1.8	1.4	1.2
BD0.9	415	467	383	719	705	689	1.7	1.5	1.8
BD1	811	499	500	1016	727	738	1.3	1.5	1.5
**48 h**									
BD0.8		998	1061		1209	1271		1.2	1.2
BD0.9		1062	1203		1287	1338		1.2	1.1
BD1		924	1171		1173	1339		1.3	1.1

**Table 3:** Melt temperatures (T_m_) of poly(ester-amide) prepolymers after 48 h reaction determined by DMA and DSC.

	Prepolymer								
	BD 0.8			BD 0.9			BD 1.0		
	AA	SA	SuA	AA	SA	SuA	AA	SA	SuA
T_m_ (°C) DMA	47.3	102.0	67.4	42.3	102.5	51.5	44.4	99.9	44.5
T_m_ (°C) DSC	27.3	103.8	32.5	34.1	105.6	45.3	40.0	103.3	44.7

**Table 4:** Thermal transitions (T_g_ and T_m_) for the various lignin-copolymers determined by DSC.

Lignin Content	BA0.8T	BA0.9T	BA1	BS0.8T	BS0.9T	BS1	BSu0.8T	BSu0.9T	BSu1
T_g1_ (°C)									
0%	−49.5	−52.4	−54.6	−25.7	−23.3	−26.5	−45.7		−24.2
10%	−46.3	−41.5	−46.6	−31.1	−19.0	−23.7	−24.2		−23.1
20%	−23.5	−49.6	−21.0	−28.8	−28.7	−23.8	−24.7	−55.0	−24.1
30%	−23.0	−40.0	−5.5	−5.8	−24.8	−25.6	−27.5	−49.4	
40%	−5.2	−21.9	−23.0	−1.2	−7.2	−23.2	−24.2	−23.7	−23.7
50%	19.6	−4.1	4.3	13.9	22.8	10.2	8.3	−6.3	−23.9
T_m_ (°C)									
0%	27.3	34.1	40.0	103.8	105.6	103.3	32.5	45.3	44.7
10%	27.2	31.9	39.3	80.3	96.4	104.7	41.5	41.5	45.6
20%	30.0	34.0	35.3	82.5	82.6	101.4	40.6	41.2	44.2
30%		29.9	37.1		78.1	94.9		39.7	41.6
T_g2_ (°C)									
10%	126.0	125.0		122.9		128.5	121.3	125.3	120.7
20%	122.9	119.7	127.2	125.2			124.4	120.3	118.4
30%	123.6	121.4	121.4	122.7	131.0	124.6	122.0	126.4	119.2
40%	122.0	120.6		125.9	123.3	126.4	123.8	125.8	123.6
50%	116.1	123.7		124.9	123.0	123.4	126.6	121.9	124.4

**Table 5:** Melt transitions (T_m_) and storage modulus (E’) at 15 °C for the various lignin-copolymers determined by DMA.

Lignin Content (%)	BA0.8T	BA0.9T	BA1	BS0.8T	BS0.9T	BS1	BSu0.8T	BSu0.9T	BSu1
T_m_ (°C)									
0	47	42	44	102	103	100	54	52	45
10	48	38	44	88	96	100	40	49	48
20	54	41	41	90	97	101	45	55	46
30	52	41	46	58	91	86	50	47	44
40	70	51	54	62	69	86	66	54	54
50	84	76	77	69	76	81	87	75	87
Storage modulus at 15 °C (MPa)									
0	195	172	332	253	316	283	371	3390	339
10	175	138	154	129	121	205	463	1600	240
20	273	181	150	120	236	233	245	344	263
30	211	192	142	945	204	59.4	394	903	207
40	239	228	200	720	440	160	314	928	77.5
50	255	167	88	520	156	257	431	791	194

**Table 6:** Major thermal degradation onset temperature (T_onset_) and T_d5_ temperature for the various lignin-copolymers determined by TGA.

Lignin Content (%)	BA0.8T	BA0.9T	BA1	BS0.8T	BS0.9T	BS1	BSu0.8T	BSu0.9T	BSu1
T_onset_ (°C)									
0	362	396	353	292	292	302	306	384	249
10	396	403	398	301	318	397	350	403	386
20	406	414	407	322	351	392	340	431	406
30	401	404	423	327	346	421	305	414	421
40	396	413	419	314	372	396	394	418	413
50	332	392	394	313	350	371	400	357	399
T_d5_ (°C)									
0	300	333	281	292	280	291	276	298	257
10	324	311	330	283	303	324	314	328	334
20	324	341	326	307	308	324	310	324	334
30	322	296	348	292	303	321	277	301	355
40	321	345	339	295	312	312	297	329	310
50	273	313	327	292	311	293	322	316	343

**Table 7:** Crystallinity (%) determined by XRD of the various lignin-co-polyester/amides.

Lignin Content (%)	BA0.8T	BA0.9T	BA1	BS0.8T	BS0.9T	BS1	BSu0.8T	BSu0.9T	BSu1
0	22	42	48	48	42	46	30	28	63
10	21	34	18	39	30	41	19	26	69
20	14	16	16	32	27	25	20	15	43
30	8	11	13	12	22	11	11	19	29
40	0	6	8	12	12	10	7	8	13
50	0	3	2	5	4	3	2	1	1

**Table 8:** Tensile properties (strength, modulus, and strain at break) of the various lignin-co-polyester/amides formulations were determined from tensile stress strain curves.

Lignin (%)	BA0.8T	BA0.9T	BA1	BS0.8T	BS0.9T	BS1	BSu0.8T	BSu0.9T	BSu1
**Tensile (Young’s) modulus (MPa)**									
10	23.6 ± 5.9	37.3 ± 5.4	52.6 ± 5.3	72.7 ± 6.9	338 ± 94	224 ± 42	38.5 ± 5.4	74.0 ± 14.2	227 ± 15
20	24.0 ± 4.0	12.8 ± 1.5	22.3 ± 2.7	77.9 ± 22.6	215 ± 58	277 ± 57	54.3 ± 14.8	111 ± 22	133 ± 18
30	10.2 ± 1.6	10.1 ± 1.0	2.3 ± 0.3	22.3 ± 6.1	102 ± 21	138 ± 29	24.4 ± 3.1	69.7 ± 18.9	40.1 ± 17.3
40	36.6 ± 8.1	17.4 ± 3.3	6.8 ± 0.8	58.1 ± 19.2	47.2 ± 11.6	93.3 ± 7.9	32.6 ± 17.6	16.4 ± 1.7	18.1 ± 1.3
50	34.9 ± 9.6	97.1 ± 6.2	59.6 ± 12.2	156 ± 78	72.3 ± 11.1	118 ± 9	113 ± 4	62.4 ± 7.8	84.5 ± 9.7
**Tensile strength (MPa)**									
10	0.093 ± 0.016	0.456 ± 0.004	0.215 ± 0.005	0.259 ± 0.043	0.482 ± 0.288	0.344 ± 0.065	0.315 ± 0.131	0.343 ± 0.167	0.469 ± 0.070
20	0.962 ± 0.271	0.277 ± 0.021	0.203 ± 0.094	0.670 ± 0.491	0.426 ± 0.123	2.11 ± 0.14	0.457 ± 0.031	0.987 ± 0.092	0.958 ± 0.158
30	0.804 ± 0.066	0.807 ± 0.010	0.265 ± 0.061	0.530 ± 0.182	1.96 ± 0.22	1.87 ± 0.09	0.644 ± 0.100	1.31 ± 0.10	1.59 ± 0.15
40	1.54 ± 0.18	0.706 ± 0.029	0.567 ± 0.125	0.844 ± 0.248	1.79 ± 0.01	1.35 ± 0.14	1.60 ± 0.45	1.30 ± 0.11	0.758 ± 0.115
50	0.734 ± 0.035	0.928 ± 0.470	0.986 ± 0.109	1.66 ± 0.11	1.06 ± 0.13	1.24 ± 0.14	0.923 ± 0.159	0.968 ± 0.075	0.871 ± 0.168
**Strain at break (%)**									
10	2.46 ± 0.31	4.70 ± 2.9	1.13 ± 0.19	0.71 ± 0.27	0.36 ± 0.07	0.30 ± 0.03	2.40 ± 1.39	1.07 ± 0.30	0.39 ± 0.03
20	17.1 ± 3.5	13.7 ± 4.0	2.43 ± 0.77	0.76 ± 0.20	0.72 ± 0.32	1.21 ± 0.31	3.42 ± 0.49	2.91 ± 0.81	1.44 ± 0.73
30	35.9 ± 5.3	72.5 ± 15.9	32.8 ± 0.6	14.3 ± 3.1	7.27 ± 1.45	2.88 ± 0.33	12.7 ± 4.6	7.97 ± 2.71	15.9 ± 4.9
40	13.6 ± 1.5	19.6 ± 5.5	29.8 ± 2.5	12.5 ± 6.5	5.68 ± 4.17	3.74 ± 1.39	22.3 ± 7.4	46.8 ± 1.4	20.7 ± 8.0
50	11.2 ± 2.0	9.07 ± 4.01	4.33 ± 1.90	4.93 ± 1.11	2.46 ± 0.60	2.60 ± 0.74	1.71 ± 0.36	7.45 ± 0.81	3.06 ± 0.75

## Data Availability

Not applicable.

## Supplementary Materials

Figure S1:Photograph showing several lignin-co-polyester/amide specimens, Figure S2: DSC thermograms showing 1st and 2nd heat cycles of BA1-10–40%lignin copolymers, Figure S3: DSC thermograms (1st heat cycle) of BA0.8T-0–50%lignin copolymers (top) and showing 1st and 2nd heat cycles of BA0.8T-0–30%lignin copolymers (bottom), Figure S4: DSC thermograms (1st heat cycle, endotherm down) of BA0.9T-0–50%lignin copolymers (top) and showing 1st and 2nd heat cycles of BA0.9T-0–30%lignin copolymers (bottom), Figure S5: DSC thermograms (1st heat cycle, endotherm down) of BS0.8T-0–50%lignin copolymers (top) and showing 1st and 2nd heat cycles of BS0.8T-0–30%lignin copolymers (bottom), Figure S6: DSC thermograms (1st heat cycle, endotherm down) of BS0.9T-0–50%lignin copolymers (top) and showing 1st and 2nd heat cycles of BS0.9T-0–30%lignin copolymers (bottom), Figure S7: DSC thermograms (1st heat cycle, endotherm down) of BS1-0–50%lignin copolymers (top) and showing 1st and 2nd heat cycles of BS1-0–30%lignin copolymers (bottom), Figure S8: DSC thermograms (1st heat cycle, endotherm down) of BSu0.8T-0–50%lignin copolymers (top) and showing 1st and 2nd heat cycles of BSu0.8T-0–30%lignin copolymers (bottom), Figure S9: DSC thermograms (1st heat cycle, endotherm down) of BSu0.9T-0–50%lignin copolymers (top) and showing 1st and 2nd heat cycles of BSu0.9T-0–30%lignin copolymers (bottom), Figure S10: DSC thermograms (1st heat cycle, endotherm down) of BSu1-0–50%lignin copolymers (top) and showing 1st and 2nd heat cycles of BSu1-0–30%lignin copolymers (bottom).

## Sample Availability

Samples of the compounds are not available from the authors.
