# Supplementary material for: Preparation and Characterization of Biobased Lignin-Co-Polyester/Amide Thermoplastics
Source: Molecules. 2021 Apr 22;26(9):2437. doi: 10.3390/molecules26092437 (PMC8122710; doi:10.3390/molecules26092437)
Supplement: Supplementary file 1 [file molecules-26-02437-s001.zip › molecules-1191046-supplementary.pdf]

Supplemental Material

Preparation and Characterization of Biobased Lignin-co-Polyester/amide Thermoplastics

Eric L. Young and Armando G. McDonald

Department of Forest, Rangeland and Fire Sciences, University of Idaho, Moscow, ID 83844, USA

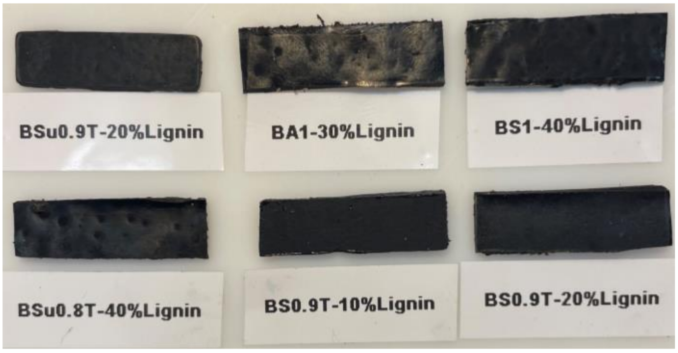

Figure S1. Photograph showing several lignin-co-polyester/amide specimens.

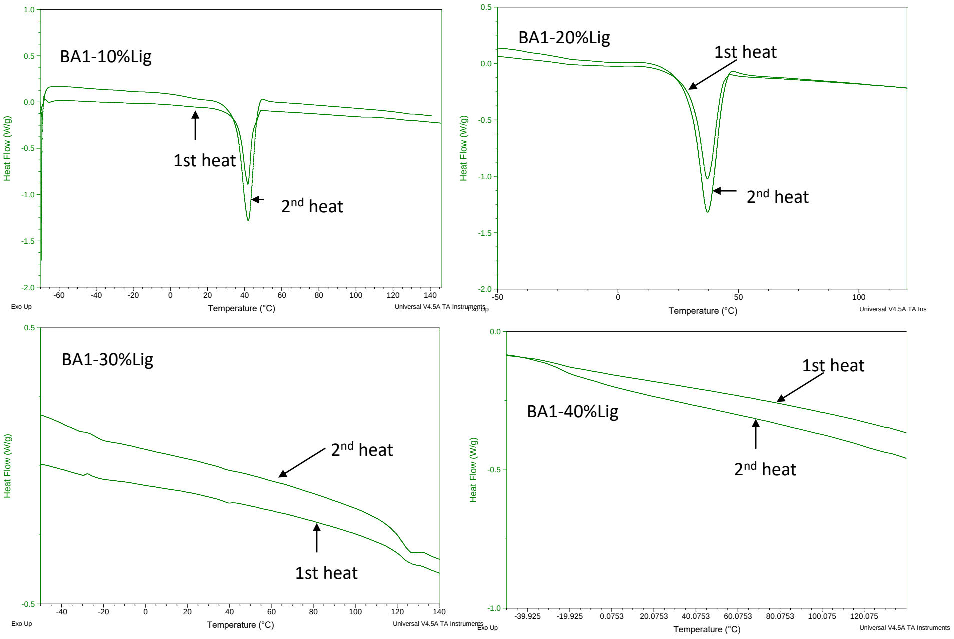

Figure S2. DSC thermograms showing 1st and 2nd heat cycles of BA1-10–40%lignin copolymers.

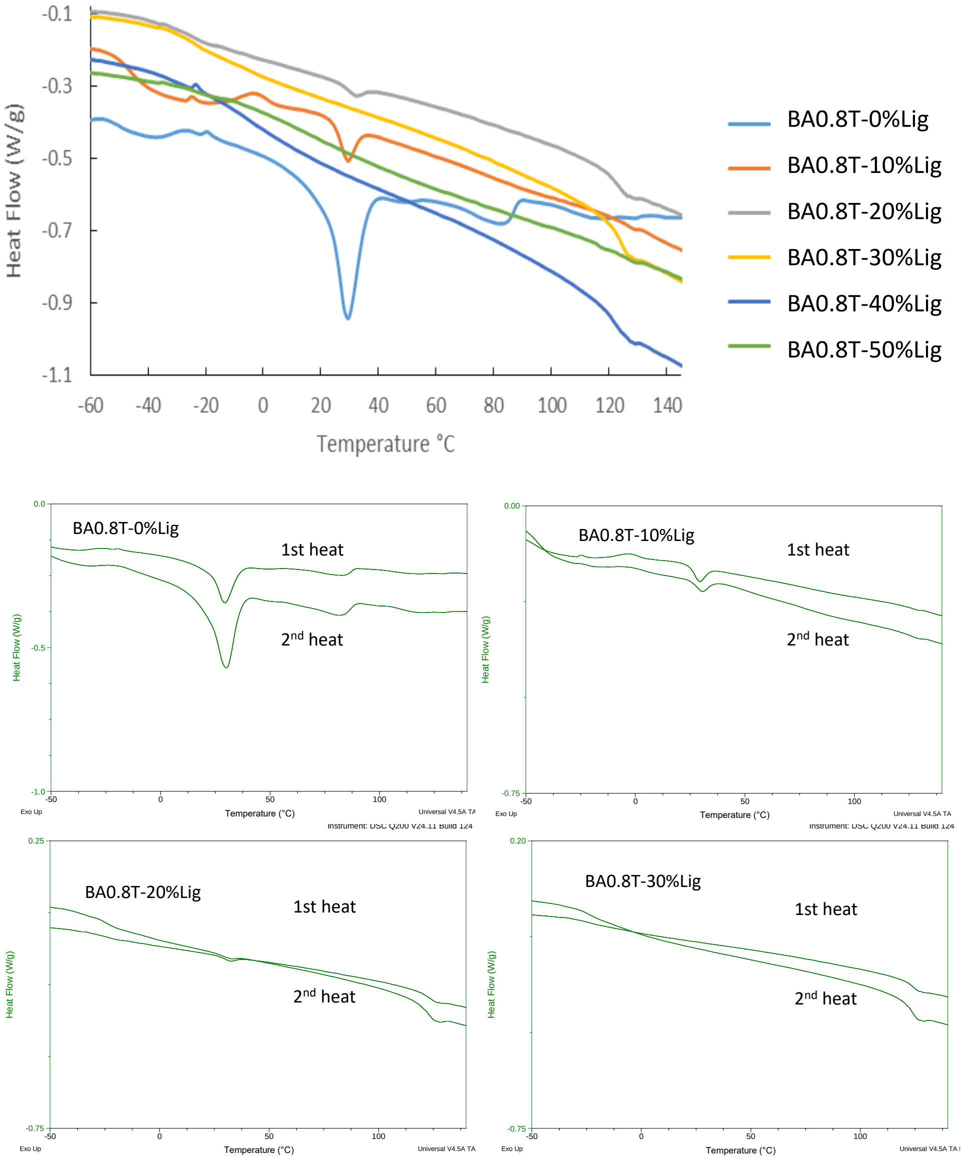

Figure S3. DSC thermograms (1st heat cycle) of BA0.8T-0–50%lignin copolymers (top) and showing 1st and 2nd heat cycles of BA0.8T-0–30%lignin copolymers (bottom).

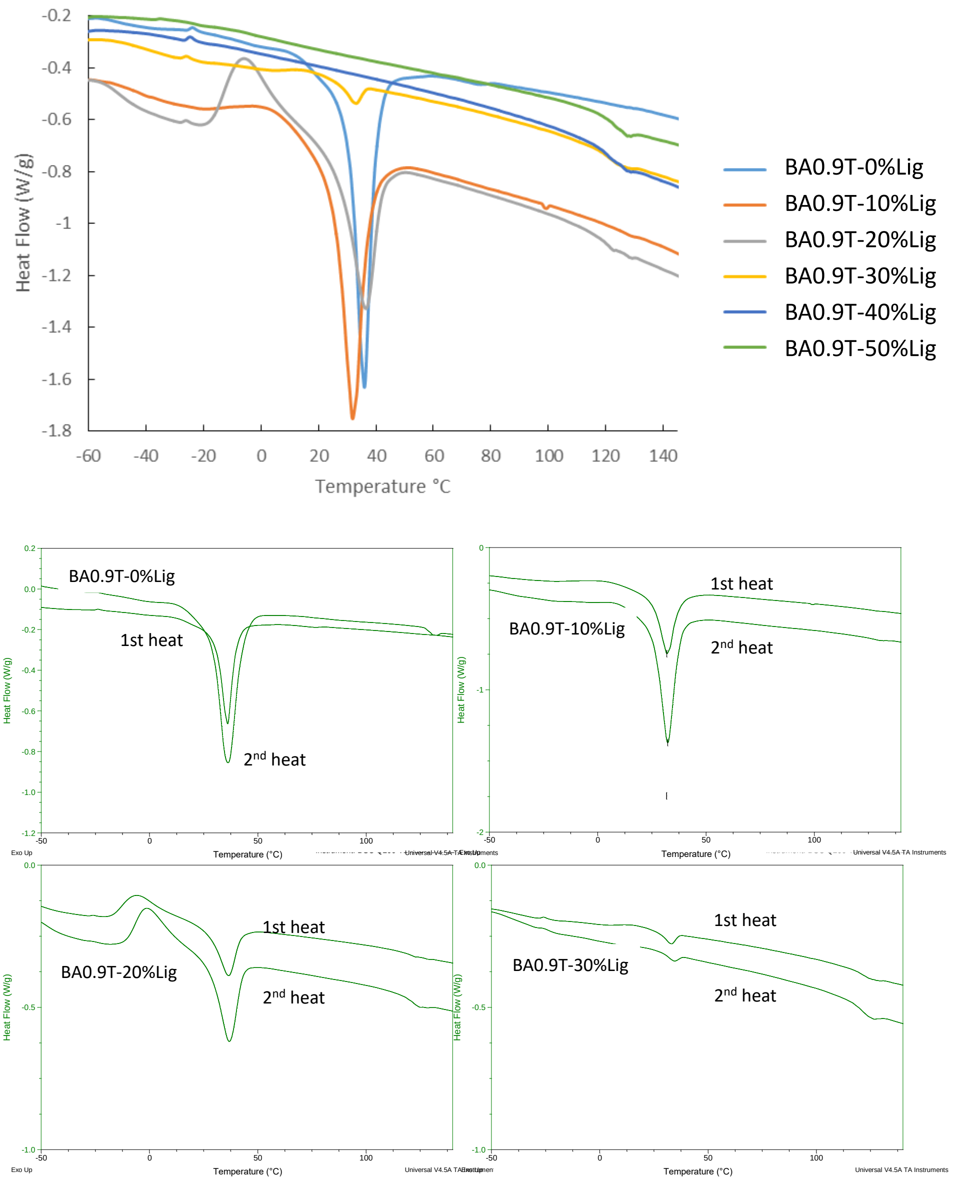

Figure S4. DSC thermograms (1st heat cycle, endotherm down) of BA0.9T-0–50%lignin copolymers (top) and showing 1st and 2nd heat cycles of BA0.9T-0–30%lignin copolymers (bottom).

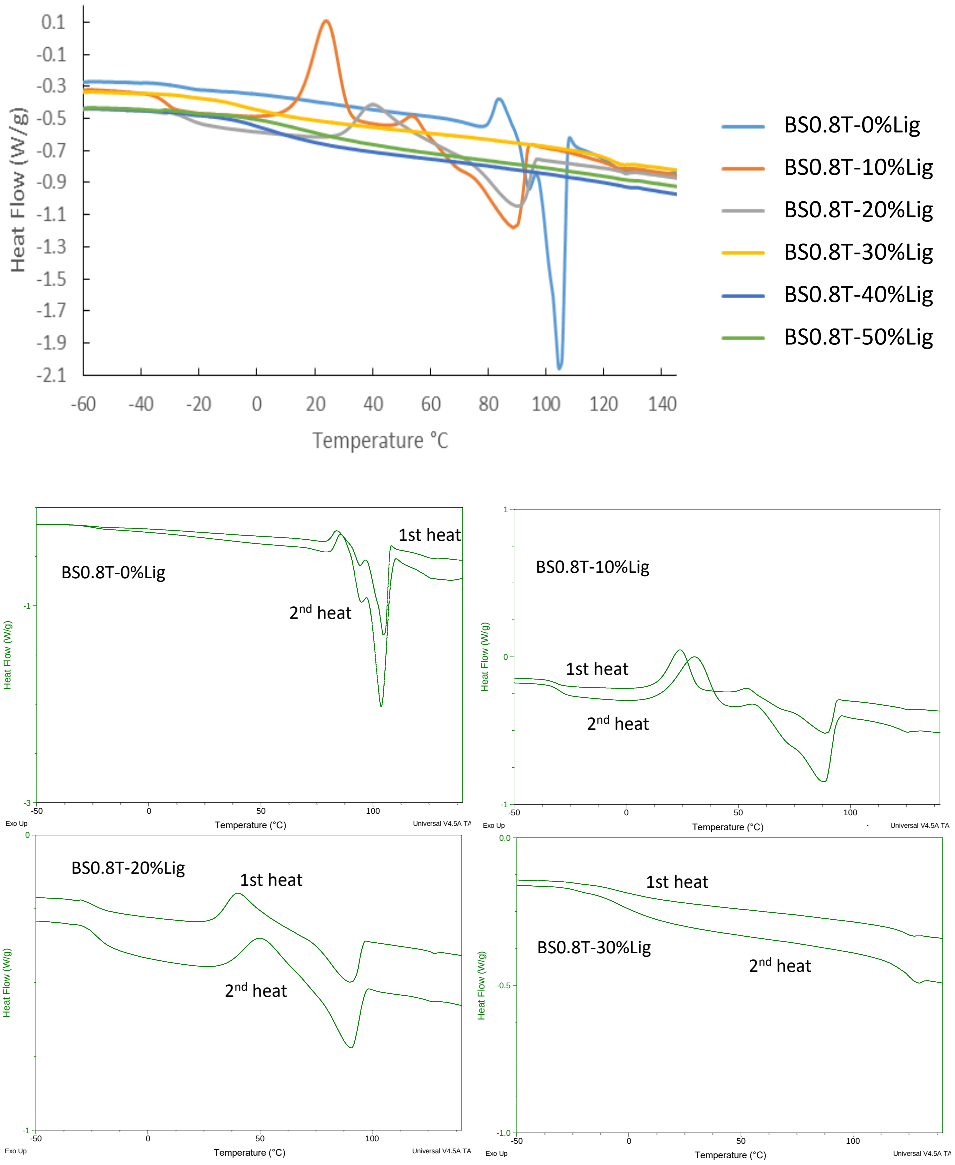

Figure S5. DSC thermograms (1st heat cycle, endotherm down) of BS0.8T-0–50%lignin copolymers (top) and showing 1st and 2nd heat cycles of BS0.8T-0–30%lignin copolymers (bottom).

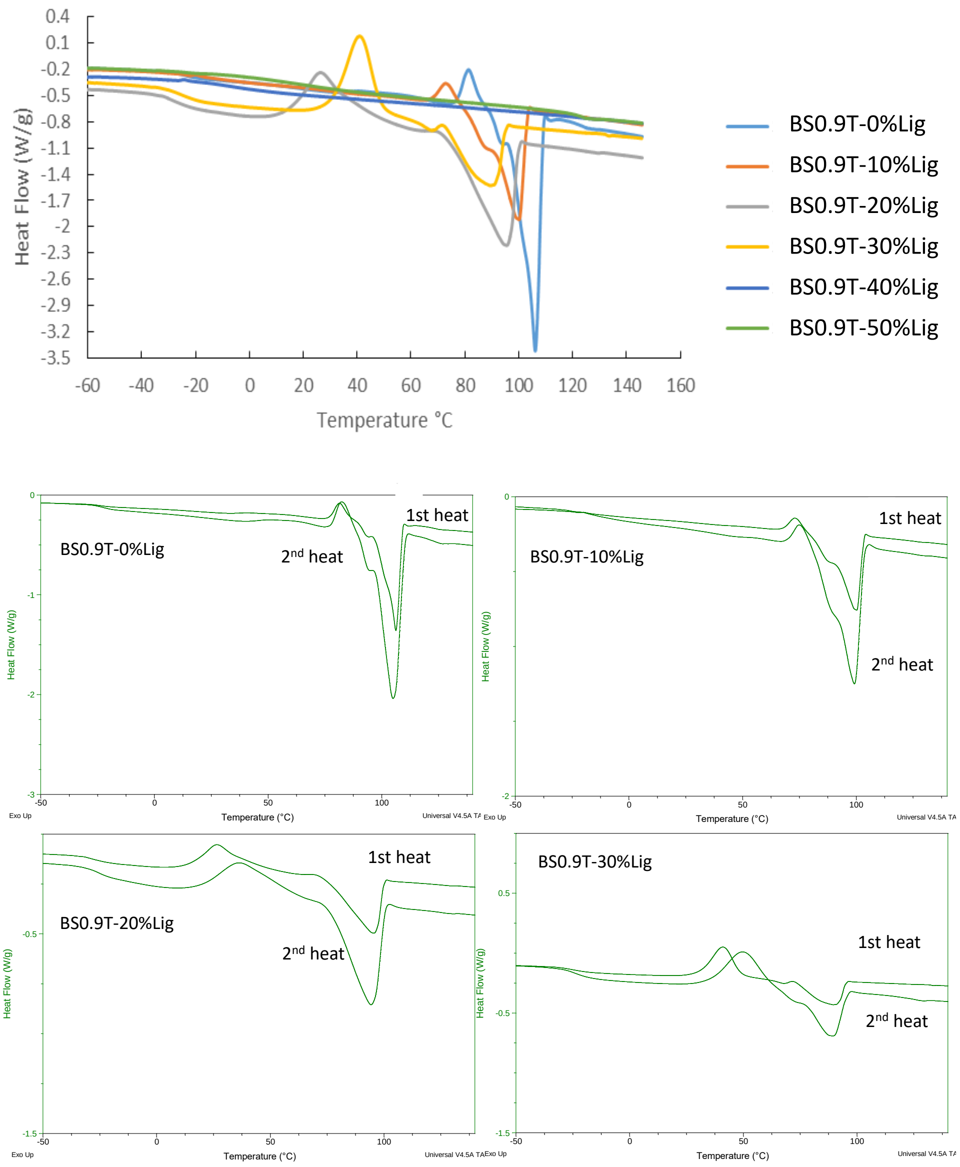

Figure S6. DSC thermograms (1st heat cycle, endotherm down) of BS0.9T-0–50%lignin copolymers (top) and showing 1st and 2nd heat cycles of BS0.9T-0–30%lignin copolymers (bottom).

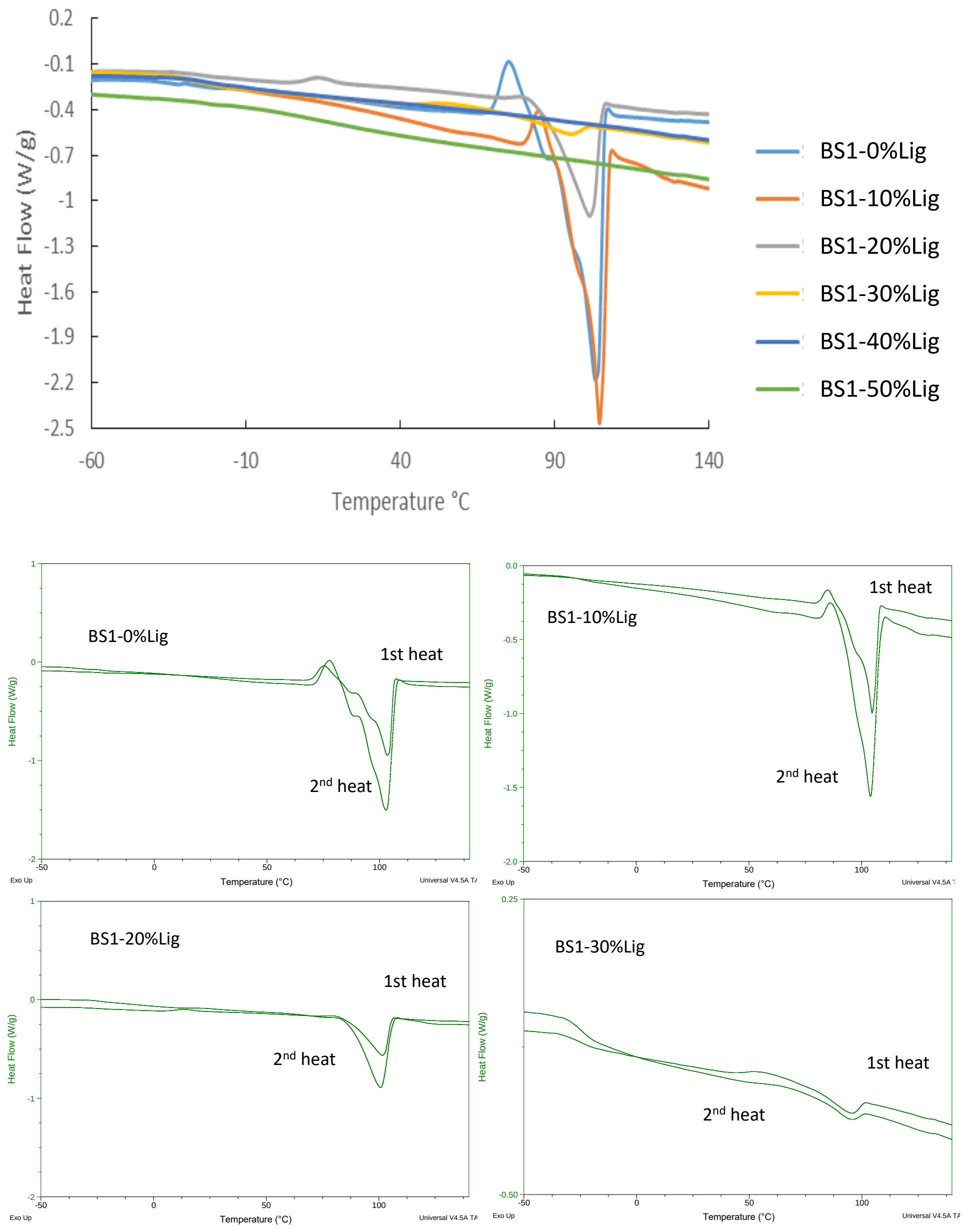

Figure S7. DSC thermograms (1st heat cycle, endotherm down) of BS1-0–50%lignin copolymers (top) and showing 1st and 2nd heat cycles of BS1-0–30%lignin copolymers (bottom).

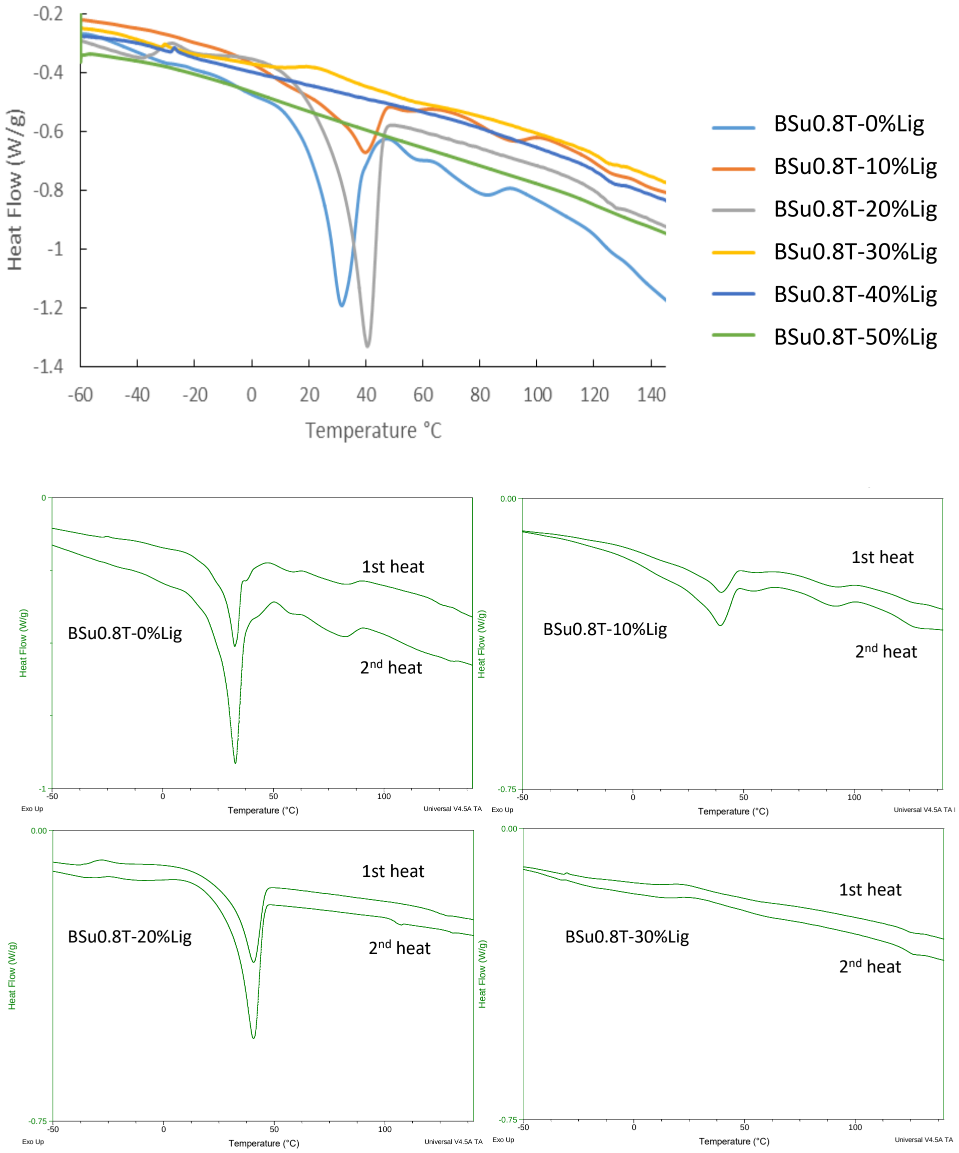

Figure S8. DSC thermograms (1st heat cycle, endotherm down) of BSu0.8T-0–50%lignin copolymers (top) and showing 1st and 2nd heat cycles of BSu0.8T-0–30%lignin copolymers (bottom).

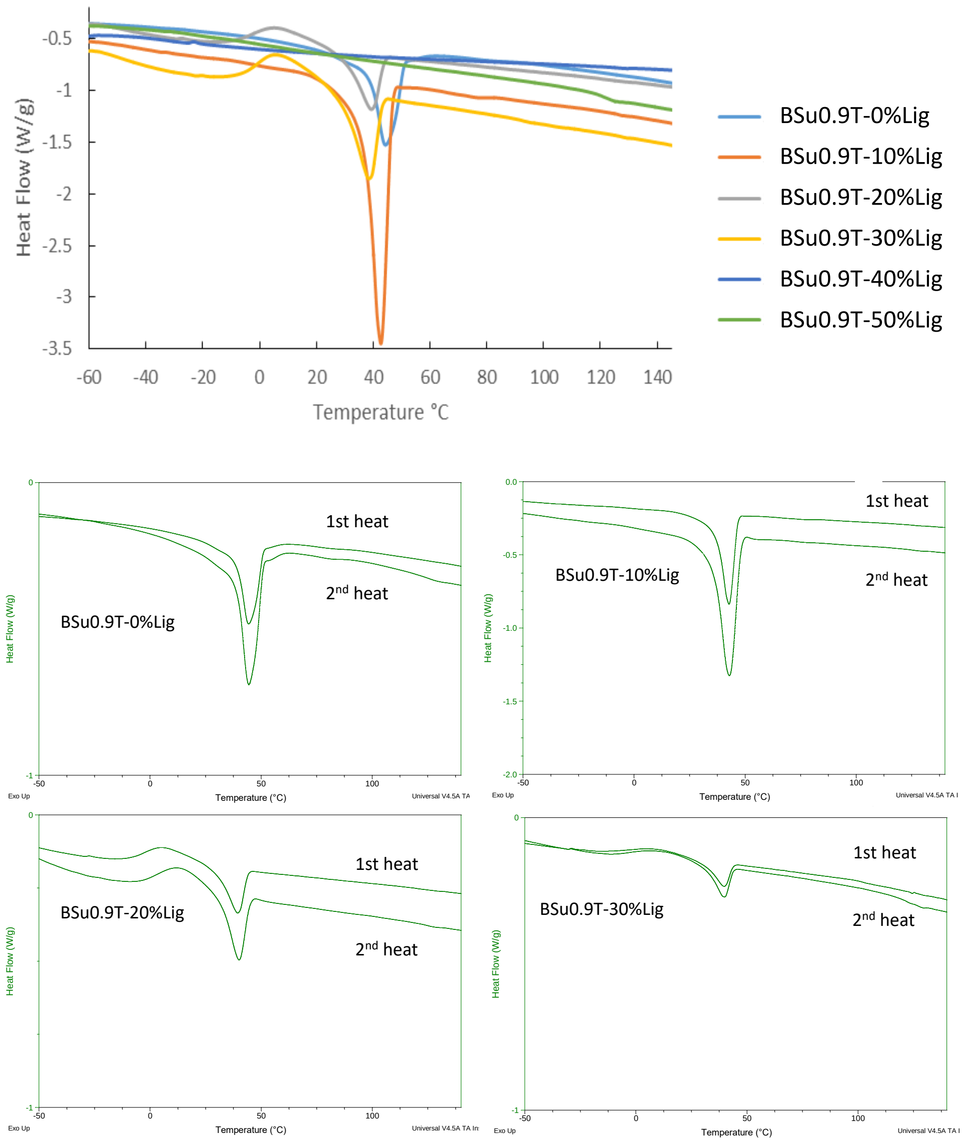

Figure S9. DSC thermograms (1st heat cycle, endotherm down) of BSu0.9T-0–50%lignin copolymers (top) and showing 1st and 2nd heat cycles of BSu0.9T-0–30%lignin copolymers (bottom).

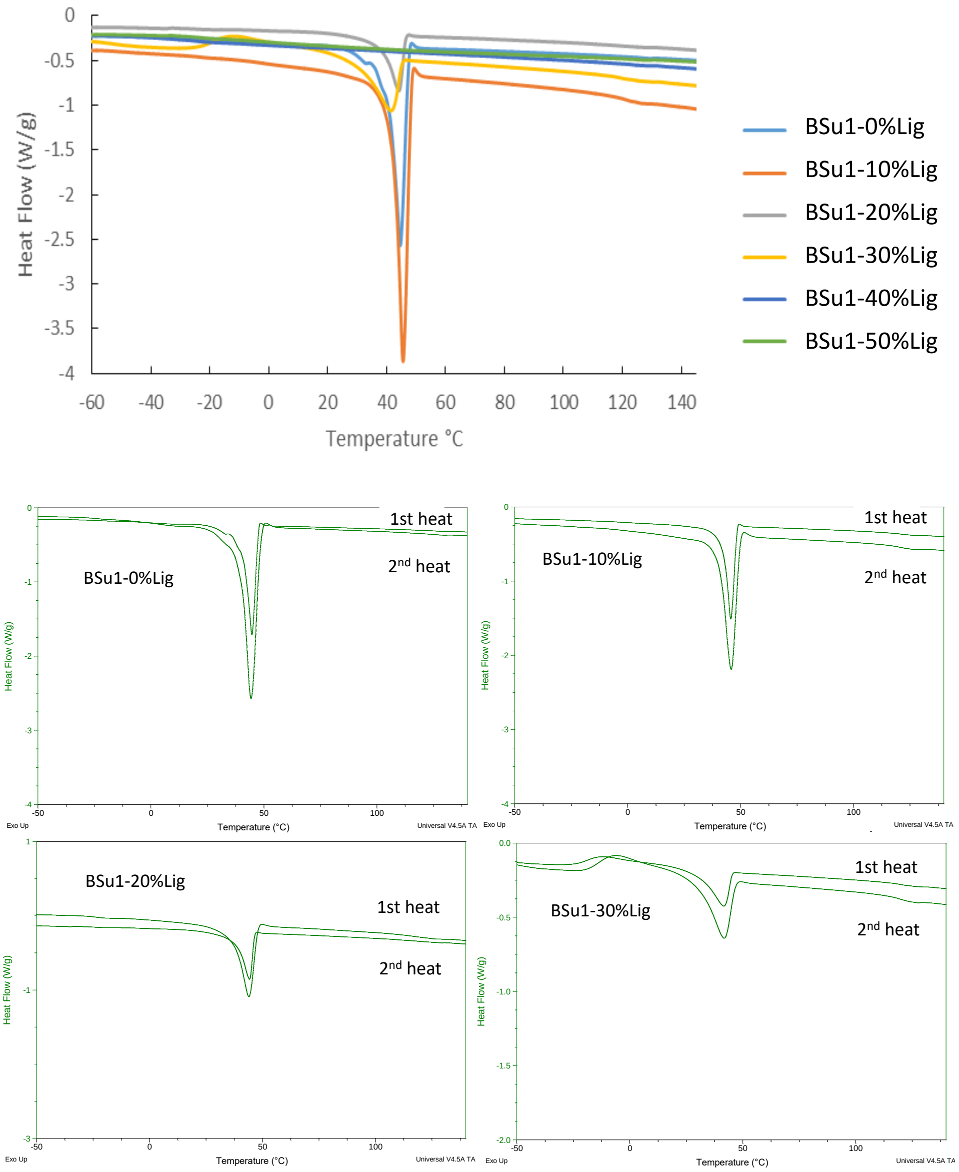

Figure S10. DSC thermograms (1st heat cycle, endotherm down) of BSu1-0–50%lignin copolymers (top) and showing 1st and 2nd heat cycles of BSu1-0–30%lignin copolymers (bottom).
